# Improve in-depth immunological risk assessment to optimize genetic-compatibility and clinical outcomes in child and adolescent recipients of parental donor kidney transplants: protocol for the INCEPTION study

**DOI:** 10.1186/s12882-021-02619-0

**Published:** 2021-12-19

**Authors:** Wai H. Lim, Brigitte Adams, Stephen Alexander, Antonia H. M. Bouts, Frans Claas, Michael Collins, Elisabeth Cornelissen, Heather Dunckley, Huib de Jong, Lloyd D’Orsogna, Anna Francis, Sebastiaan Heidt, Jean Herman, Rhonda Holdsworth, Joshua Kausman, Rabia Khalid, Jon Jin Kim, Siah Kim, Noël Knops, Vasilis Kosmoliaptsis, Cynthia Kramer, Dirk Kuypers, Nicholas Larkins, Suetonia C. Palmer, Chanel Prestidge, Agnieszka Prytula, Ankit Sharma, Meena Shingde, Anne Taverniti, Armando Teixeira-Pinto, Peter Trnka, Francis Willis, Daniel Wong, Germaine Wong

**Affiliations:** 1 Department of Renal Medicine, Sir Charles Gairdner Hospital, Perth, Western Australia 6009 Australia; 2 Medical School, University of Western Australia, Perth, Australia; 3 Department of Pediatric Nephrology, Hôpital Universitaire des Enfants-Reine Fabiola, Brussels, Belgium; 4 Department of Nephrology, Westmead Children’s Hospital, Sydney, Australia; 5 Faculty of Medicine and Health, University of Sydney, Sydney, Australia; 6 Department of Pediatric Nephrology, Emma Children’s Hospital, Amsterdam University Medical Center, Amsterdam, The Netherlands; 7 Department of Immunology, Leiden University Medical Centre, Leiden, The Netherlands; 8 Department of Renal Medicine, Auckland District Health Board, Auckland City Hospital, Auckland, New Zealand; 9 Department of Medicine, Faculty of Medical and Health Sciences, University of Auckland, Auckland, New Zealand; 10 Department of Pediatric Nephrology, Radboud University Medical Center, Amalia Children’s Hospital, Nijmegen, The Netherlands; 11 New Zealand Transplantation and Immunogenetics Laboratory, New Zealand Blood Service, Auckland, New Zealand; 12 Erasmus Medical Center, Rotterdam, The Netherlands; 13 Faculty of Health and Medical Sciences, Pathology & Laboratory Medicine, University of Western Australia, Perth, Australia; 14 Department of Clinical Immunology, Fiona Stanley Hospital, Perth, Australia; 15 National Pancreas Unit, Westmead Hospital, Sydney, Australia; 16 School of Medicine, University of Queensland, Brisbane, Queensland Australia; 17 Child and Adolescent Renal Service, Children’s Health Queensland, Brisbane, Queensland Australia; 18 Department of Pediatric Nephrology and Solid Organ Transplantation, University Hospitals Leuven, Leuven, Belgium; 19 Victoria Transplant and Immunogenetics Service, Australian Red Cross Blood Service, West Melbourne, Victoria Australia; 20 Department of Nephrology, Royal Children’s Hospital Melbourne, Parkville, Victoria Australia; 21 Murdoch Children’s Research Institute, Melbourne, Victoria Australia; 22 University of Melbourne, Melbourne, Victoria Australia; 23 Sydney School of Public Health, The University of Sydney, Sydney, Australia; 24 Centre for Kidney Research, The Children’s Hospital at Westmead, Westmead, Sydney, Australia; 25 Department of Paediatric Nephrology, Nottingham University Hospital, Nottingham, UK; 26 Department of Paediatric Nephrology, Great Ormond Street Hospital for Children NHS Foundation Trust, London, WC1N 3JH UK; 27 MRC Centre for Transplantation, Guy’s Hospital, London, UK; 28 Department of Development and Regeneration (Woman and Child), KU Leuven, Leuven, Belgium; 29 Department of Surgery, University of Cambridge, Addenbrooke’s Hospital, Hills Road, Cambridge, UK; 30 UK and NIHR Blood and Transplant Research Unit in Organ Donation and Transplantation, University of Cambridge, Hills Road, Cambridge, UK; 31 NIHR Cambridge Biomedical Research Centre, Hills Road, Cambridge, UK; 32 Department of Nephrology and Renal Transplantation, University Hospitals Leuven, Leuven, Belgium; 33 Department of Microbiology and Immunology, KU Leuven, University of Leuven, Leuven, Belgium; 34 Department of Nephrology, Perth Children’s Hospital, Perth, Australia; 35 University of Otago Christchurch, Christchurch, New Zealand; 36 Starship Children’s Hospital, Auckland Health Board, Auckland, New Zealand; 37 Department of Pediatric Nephrology and Rheumatology, Ghent University Hospital, Ghent, Belgium; 38 Department of Renal Medicine, Westmead Hospital, Sydney, Australia; 39 NSW Health Pathology, Institute of Clinical Pathology and Medical Research, Westmead Hospital, Westmead, Sydney, Australia; 40 School of Medicine, University of Notre Dame (Fremantle), Perth, Australia; 41 Anatomical Pathology, PathWest, QEII Medical Centre, Perth, Australia

**Keywords:** Kidney transplant, Children, Adolescents, Parental donor, Immunological profile, Human leukocyte antigen, Antibody, Rejection, Allograft loss

## Abstract

**Background:**

Parental donor kidney transplantation is the most common treatment option for children and adolescents with kidney failure. Emerging data from observational studies have reported improved short- and medium-term allograft outcomes in recipients of paternal compared to maternal donors. The INCEPTION study aims to identify potential differences in immunological compatibility between maternal and paternal donor kidneys and ascertain how this affects kidney allograft outcomes in children and adolescents with kidney failure.

**Methods:**

This longitudinal observational study will recruit kidney transplant recipients aged ≤18 years who have received a parental donor kidney transplant across 4 countries (Australia, New Zealand, United Kingdom and the Netherlands) between 1990 and 2020. High resolution human leukocyte antigen (HLA) typing of both recipients and corresponding parental donors will be undertaken, to provide an in-depth assessment of immunological compatibility. The primary outcome is a composite of de novo donor-specific anti-HLA antibody (DSA), biopsy-proven acute rejection or allograft loss up to 60-months post-transplantation. Secondary outcomes are de novo DSA, biopsy-proven acute rejection, acute or chronic antibody mediated rejection or Chronic Allograft Damage Index (CADI) score of > 1 on allograft biopsy post-transplant, allograft function, proteinuria and allograft loss. Using principal component analysis and Cox proportional hazards regression modelling, we will determine the associations between defined sets of immunological and clinical parameters that may identify risk stratification for the primary and secondary outcome measures among young people accepting a parental donor kidney for transplantation. This study design will allow us to specifically investigate the relative importance of accepting a maternal compared to paternal donor, for families deciding on the best option for donation.

**Discussion:**

The INCEPTION study findings will explore potentially differential immunological risks of maternal and paternal donor kidneys for transplantation among children and adolescents. Our study will provide the evidence base underpinning the selection of parental donor in order to achieve the best projected long-term kidney transplant and overall health outcomes for children and adolescents, a recognized vulnerable population.

**Trial registration:**

The INCEPTION study has been registered with the Australian New Zealand Clinical Trials Registry, with the trial registration number of ACTRN12620000911998 (14th September 2020).

## Background

Kidney transplantation is the treatment of choice for people with kidney failure, conferring a significant survival advantage compared to people treated with long-term dialysis [1]. Live donor kidney transplantation is preferred because survival rates of patients and allografts are substantially longer in comparison with deceased donor transplants [2–4]. In addition, living donation facilitates pre-emptive transplantation, avoiding the deleterious effects on health, psychosocial and educational outcomes of dialysis treatment on children during critical periods of growth and development [3].

Most pediatric and adolescent kidney transplant recipients require subsequent transplants and following allograft failure are often highly sensitized (with development of multiple anti-human leukocyte antigen [HLA] antibodies that may hinder future transplant potential) and at higher risk of death [5]. Selecting the donor kidney associated with the best possible long-term allograft outcome is vital. Parental donors account for over 60% of live donor kidneys for pediatric and adolescent patients with kidney failure in the United Kingdom, Australia and New Zealand [6, 7]. However, it is unclear whether maternal versus paternal donation is associated with differential allograft outcome. A recent population cohort study showed that kidney transplants from maternal donor kidneys were associated with an up to a 60% greater risk of acute rejection and allograft loss compared to paternal donor kidneys [8]. This finding is contradictory to the previously held paradigm that exposure to non-inherited maternal antigen (NIMA) may incite a lesser immunological response compared to exposure to non-inherited paternal antigen (NIPA). Previous cohort studies and experimental models have suggested that exposure of a child to the NIMA during pregnancy may lead to NIMA-specific tolerance. In one study, sibling transplants expressing NIMA were associated with lower risk of rejection and superior allograft outcomes compared to transplants expressing the NIPA [9–11]. Given that parental donors are the predominant source of live donor kidneys for pediatric and adolescent patients with kidney failure, this study will help inform whether HLA matching at the epitope, amino acid and physiochemical properties level may enhance the understanding of the differential antigenicity and immunogenicity of NIMA and NIPA on allograft outcomes following parental donor kidney transplantation. The findings from this study may potentially have important clinical implications in the selection of the appropriate parental donor kidney for transplantation.

The over-arching objective of this study is to address the current knowledge gap of the “NIMA paradox” by identifying potential differences in immunological compatibility between maternal and paternal donor kidneys and how this compatibility affects allograft outcomes. The study aims include:

(i) Detailed immunological profiling for pediatric and adolescent recipients of parental donor kidneys with and without adverse allograft outcomes of the development of de novo donor-specific anti-HLA antibody (DSA), any acute rejection or allograft loss;
(ii) Determine the association between different immunological risk profiles and adverse allograft outcomes; and
(iii) Determine whether the associations between the immunological risk profiles and adverse allograft outcomes are modified by parental status and other clinical factors.

## Methods/design

### Study design and setting

**Fig. 1:**
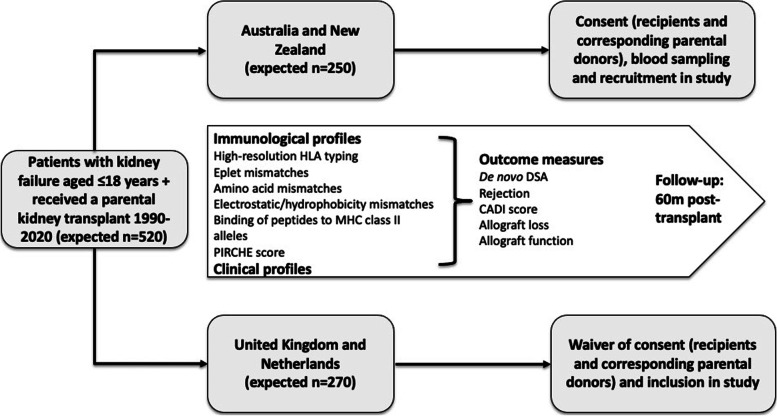
Design, participants and outcome measures of the INCEPTION study

The INCEPTION study is a longitudinal observational study that will recruit pediatric and adolescent transplant recipients aged ≤18 years who have received parental donor kidneys between January 1990 and December 2020 (Fig. 1). Corresponding parental donors will also be recruited for inclusion in the study. Adult kidney transplant recipients of parental donor kidneys aged > 18 years at time of transplantation or recipients of deceased donor kidney transplants will be excluded. Funding for the INCEPTION study is provided by the National Health and Medical Research Council (NHMRC) Ideas Grant (Application ID: APP1184595, funding duration 2020-2023), the Department of Health Western Australia and the Telethon-Perth Children’s Hospital Research Fund (funding duration 2017-2020) and Starship Foundation Clinical Research Grant (Auckland, New Zealand). The reporting and conduct of the study will adhere to The Strengthening the Reporting of Observational studies in Epidemiology (STROBE) guidelines [12].

This study will be conducted across 4 countries including Australia (5 transplant centres), New Zealand (1 transplant centre), United Kingdom (UK, 13 transplant centres) and the Netherlands (3 transplant centres). The INCEPTION study has been registered with the Australian New Zealand Clinical Trials Registry (ANZCTR) prior to recruitment, with the trial registration number of ACTRN12620000911998 (registration approved 14th September 2020).

### Study recruitment and data capture

In Australia and New Zealand, parental donor-recipient pairs that fulfil the inclusion criteria will be identified through the Australia and New Zealand Dialysis and Transplant (ANZDATA) registry and forwarded to local site investigators. Only donor-recipient pairs recorded in the registry as living and residing in Australia and New Zealand will be approached for participation. Each donor-recipient pair will provide written or verbal informed consent or age-appropriate assent by the site study investigators or delegates before participation. The Sir Charles Gairdner Osborne Park Health Care Group Human Research Ethics Committee (Perth, Western Australia, Australia) approved the research application in December 2018, with reciprocal ethics approvals covering all participating sites in Australia and New Zealand. Consent for New Zealand participation was obtained from the New Zealand Health and Disability Ethics Committee (Ministry of Health, Wellington, New Zealand). Verbal consent will be utilized during the current pandemic of COVID-19 infection, with the HREC of each participating site granting the approval to this amendment if appropriate. In the United Kingdom (approval from the United Kingdom National Health Service [NHS] in England and Wales) and the Netherlands (approval from the Medical Ethics Review Committee of the Amsterdam Academic Medical Center), project-specific waiver of consents for serum or deoxyribonucleic acid (DNA) biobanking and conduct of research studies relating to kidney transplant outcomes have been granted, and the conduct of this study have received updated ethics approvals.

### Study procedure

In Australia and New Zealand, all consented donor-recipient pairs will attend a single study visit to have 20 mL of blood drawn for separation into serum/plasma and deoxyribonucleic acid (DNA) isolation. This blood sampling will be taken from all donor-recipient pairs regardless of the availability of prior stored serum/plasma and DNA for testing. The timing of this additional blood test for recipients will coincide with their routine clinical blood withdrawal where possible. Donor and recipients will also consent to allow access of their health records from ANZDATA registry (or equivalent healthcare registries in other countries), Organ Matching (Australia-specific) and other country-specific Blood Service systems (a computer data system that stores information relating to organ allocation and donor kidney details, including information on donor and recipient HLA typing and pre-transplant and de novo DSA) and local health records from their respective hospitals. For donor-recipient pairs in the UK and the Netherlands, the ethics approval will allow for the utilization of the stored serum/plasma and DNA for testing (where required), with no additional blood sampling to be requested from these pairs.

### Defining detailed donor-recipient immunological risk profiles

#### High resolution HLA typing

Over the last decade, HLA-typing has evolved from serological typing to high resolution molecular typing covering HLA-A, −B, −C, −DRB1/3/4/5, −DPA1, −DPB1, −DQA1 and -DQB1 loci, providing a more comprehensive and accurate assessment of tissue compatibility in transplantation. High-resolution molecular HLA typing for the 11 HLA-loci will be performed using a Next Generation Sequencing (NGS) or an alternative high-resolution typing technique by the respective tissue typing laboratories in each country. Given NGS HLA-typing across all HLA alleles is considered standard practice for living donor transplantation in many transplanting centers, re-typing of donors/recipients will not be undertaken unless typing across all HLA alleles at the high-resolution level is not available.

#### Quantifying HLA-eplet mismatches

Epidemiological studies have consistently shown an association between an increasing number of epitope or eplet mismatches and an increased risk of adverse allograft outcomes, including the development of de novo DSA, antibody mediated rejection (AMR), transplant glomerulopathy (TG) and/or premature allograft loss [13, 14]. High-resolution molecular typing provides the opportunity for a more comprehensive and accurate assessment of HLA compatibility in transplantation. However, compatibility based on the many thousands of HLA alleles is fundamentally impossible, but the variation in HLA alleles can be explained by differences in a relatively low number of epitopes, which are part of the HLA protein, made up of polymorphic amino acid residues. Eplets are short discontinuous sequences of amino acid residues within each HLA epitope that can theoretically elicit a B-cell driven immune response in the recipients (immunogenicity) [15, 16]., Therefore, compatibility assessment focusing on eplet mismatches, calculated using a computer program known as HLAMatchmaker [16, 17], is likely to have the potential to improve prediction of adverse allograft outcomes [18]. The identification and quantification of the respective location and number of eplet mismatches at HLA class I (HLA-A, −B, −C) and class II (HLA-DRB1/3/4/5, −DPA1, −DPB1, −DQA1 and -DQB1) loci will be determined by entering the 2-field molecular HLA typing for each donor and recipient pair into the HLAMatchmaker to enable a comparison of the HLA eplet-profiles of each donor/recipient pair (HLA-ABC v2.0 and HLA-DRDQDP v2.1; http://www.eplets.net).

#### Donor-recipient amino acid polymorphisms and physicochemical HLA incompatibility

Eplets are theoretically defined (combinations of) amino acids that have been subject to change, making clinical integration of this approach difficult [19–21]. Assessment of the physicochemical disparity between donor and recipient HLA, in addition to comparing the interlocus and intralocus amino acid polymorphisms in antibody-accessible regions of the HLA molecules may improve risk stratification and predict adverse outcomes post-transplant [22–24]. The specificity and stability of antigen-antibody binding is determined by the structural compatibility and the electrostatic and hydrophobic interactions between the two molecules [25]. Donor and recipient HLA class I and class II mismatches will be compared at the sequence level to derive the number and physicochemical disparity of amino acid polymorphisms on donor HLA molecules using the Cambridge HLA immunogenicity algorithm [22–24]. The solvent accessibility of these amino acid polymorphisms will also be assessed using the HLA Epitope Mismatch Algorithm (EMMA; https://hla-emma.com) and Cambridge HLA immunogenicity algorithm [24, 26]. The electrostatic dissimilarity between donor and recipient HLA molecules at the structural level will be assessed using the recently described electrostatic mismatch score (EMS-3D) [27]. These scores may provide additional predictive value for class I and II allo-antibody responses but the clinical applicability of one or more of these scores remains unclear [24, 27].

#### Quantifying the role of T-cell help in DSA development

Until recently, the contribution of T cells to DSA development was not quantifiable. T-cell help is essential in the development of immunoglobulin (Ig)-G DSA by promoting the differentiation and proliferation of antigen-specific naïve B cells into memory B-cells and plasma cells. Peptides derived from donor HLA molecules are presented by HLA class II molecules of recipient B cells to cognate helper T cells, which then provide signals and cytokines to these B cells to undergo affinity maturation, class switching and differentiation into plasma cells [28]. We will utilize the NetMHCIIpan software (www.cbs.dtu.dk/services/NetMHCII-2.3 and www.cbs.dtu.dk/services/NetMHCIIpan-3.2) [29] to determine the pan-specific binding of peptides to MHC class II alleles of known sequence using pre-specified affinity thresholds and peptide ranking and other approaches in development by the study team [22, 23, 30]. In addition, we will also utilize the predicted indirectly recognizable HLA epitopes (PIRCHE) computational algorithm, which is designed to predict indirectly recognizable HLA-derived donor peptides that may induce T-cell allorecognition and lead to the production of donor-specific anti-HLA IgG antibodies (https://www.pirche.com/pirche/#/features/bioinformatics) to quantify the “amount” of T- cell help [31]. A high number of PIRCHE, likely to represent a higher number of “theoretical” T-cell epitopes that can be presented by recipient HLA class II molecules, may correlate with clinical alloreactivity but the clinical significance of PIRCHE on long-term allograft outcomes is yet to be determined.

### Outcome measures

The primary outcome is the composite of de novo DSA (defined as mean fluorescence intensity [MFI] of over 500), or any biopsy-proven acute rejection or allograft loss (defined as returning to dialysis or death with functioning allograft) after parental donor kidney transplantation up to 60-months post-transplant. Secondary outcomes are individual components of the primary composite outcome (development of de novo DSA, any biopsy-proven acute rejection, allograft loss at 60-months post-transplant but also extended follow-up to 120-months post-transplant), acute or chronic AMR (including the presence of TG) or Chronic Allograft Damage Index (CADI) score of > 1 on allograft biopsy post-transplant, allograft function (creatinine-derived estimated glomerular filtration rate [eGFR] using the Chronic Kidney Disease-Epidemiology Collaboration [CKD-EPI] equation for recipients aged ≥16 years [32] or bedside Schwartz equation for recipients aged < 16 years [33–35]), and urine proteinuria.

#### De novo DSA

Post-transplant sera of all recipients are tested pre-transplant, when clinically indicated or routinely at pre-specified time-points post-transplant (in some centres) for de novo DSA directed against either the maternal or paternal donor HLA across HLA-A, −B, −C, −DRB1, −DRB3/4/5, −DPA1, −DPB1, −DQA1 and -DQB1 alleles. In brief, an aliquot of a single antigen bead (SAB) mixture will be incubated with a small volume of patients’ sera (approximately 50 μl) containing the anti-HLA antibody as per manufacturer’s instructions. The analysis will be undertaken on a Luminex (or equivalent) platform and the reactivity will be determined with the manufacturer’s software and expressed as mean fluorescence intensity (MFI) for each mismatched HLA. Antibodies to these HLA alleles with MFI of varying thresholds (from MFI ≥ 500) will be considered a “positive result” because we and others have shown a consistent association between the presence of pre-transplant DSA with MFI ≥500 and a heightened risk of rejection after kidney transplantation [36, 37]. The MFI threshold value of ≥500 is defined using the Luminex platform. The time-points for the monitoring and testing for pre-transplant and de novo DSA will be undertaken according to the standard policies of each participating site; typically, pre-transplant, annually post-transplant and/or when clinically indicated (during episodes of allograft dysfunction or acute rejection).

#### Assessment for acute rejection and CADI

Episodes of biopsy-proven acute and chronic rejection (diagnosed on protocol or clinical indication biopsies) post-transplant will be assessed and reported according to the validated Banff classification [38]. Consistent with this, each rejection episode will be categorized as acute, chronic or acute on chronic; and further stratified to cellular, vascular, antibody mediated or mixed pattern rejections. The most severe type of rejection in those with a mixed pattern will be considered the dominant lesion (i.e. vascular or antibody-mediated > cellular rejections). TG (Banff cg 1-3) will be coded according to the Banff classification and is considered a morphological manifestation of chronic AMR [39].

The CADI score quantifies the amount of chronic damage to the allograft, with the score calculated from a total of six parameters of: (a) diffuse or focal inflammation and (b) fibrosis in the interstitium, (c) mesangial matrix increase and (d) sclerosis in glomeruli, (e) intimal proliferation of vessels, and (f) tubular atrophy; with each individual parameter being scored between 0 and 3 as described in other studies [40]. The CADI score is a global standardized scoring system based on pre-defined criteria and is considered part of standard reporting in all pathology laboratories. Previous studies have consistently shown that an elevated CADI score correlates with acute rejection as well as chronic rejection and allograft loss up to 4 years post-transplant, indicating that CADI score has important prognostic significance [41–45].

Renal allograft biopsies (protocol or clinical indication biopsies) are frequently undertaken after kidney transplantation and are typically prepared into formalin-fixed paraffin-embedded tissue blocks or stored as frozen tissues. Representative images or whole slide imaging (using digital microscopy or site-specific systems such as the Sysmex system according to site availability) for histological scoring to capture the abnormal findings will be requested from each histopathology department, which will comprise of at least one Periodic acid–Schiff (PAS) and one Trichrome-stained images (where possible). If multiple biopsy specimens were available in the first 12 months post-transplant, only tissues from two of these biopsies (one between 0 and 6 months and the other between > 6-12 months if available) will be imaged. A nominated blinded pathologist will re-score the stored digital images (where available) in accordance with the established CADI parameters and Banff criteria for rejection and chronicity [41, 46].

### Clinical and laboratory parameters

In addition to the immunological profiling and outcome measures, a number of donor and recipient characteristics will be extracted from registries, databases and local hospital healthcare records where appropriate and available. Data to be extracted include donor factors of age, sex, body mass index, donor relationship to recipient, comorbid conditions and race; recipient factors of age, sex, body mass index, race, socio-economic status (SES; derived from post-code in Australia or alternative SES parameters in other countries), prior episodes of non-adherence to immunosuppressive agents or missed appointments (as recorded in healthcare records), comorbid conditions (including complex syndrome, urological, liver or cardiac diseases) and dialysis duration; and transplant-related factors of transplant era, ischaemic time, sensitization status of percentage class I and II panel reactive antibody (%PRA), types and intra-patient variability of the calcineurin-inhibitor levels; all of which are known to be associated with the clinical outcomes of interest. Information relating to measures of potential sensitization to maternal antigens, including complications in maternal pregnancy including infections, breast-feeding and prior pregnancies will be sought from respective donors (where available).

Specific outcome measures include acute rejection (and Banff classification) at any time point post-transplant (types, severity and response to treatment), allograft function (creatinine and eGFR at 3 and 6 months, then annually post-transplant), allograft failure (including causes of allograft failure) and death (including causes of death) will be extracted from ANZDATA registry or relevant sources in other countries; and kidney biopsy reports, rejection-related treatment and response to treatment, intensity of immunosuppression (therapeutic drug levels for calcineurin inhibitor [CNI] at 3, 6, 12, 24, 36, 48, and 60 months post-transplant), proteinuria (urine protein/creatinine ratios at 3, 6, 12, 24, 36, 48, and 60 months post-transplant if available) and number/duration of all-cause and cause-specific hospitalisations will be extracted from local hospital and laboratory data. The data will be entered into a password-protected database (unique to each site) at least up to 5-years post-transplant (or until allograft loss up to 10-years post-transplantation using linkage data to ANZDATA and other country-specific registries).

### Sample size calculation

**Fig. 2:**
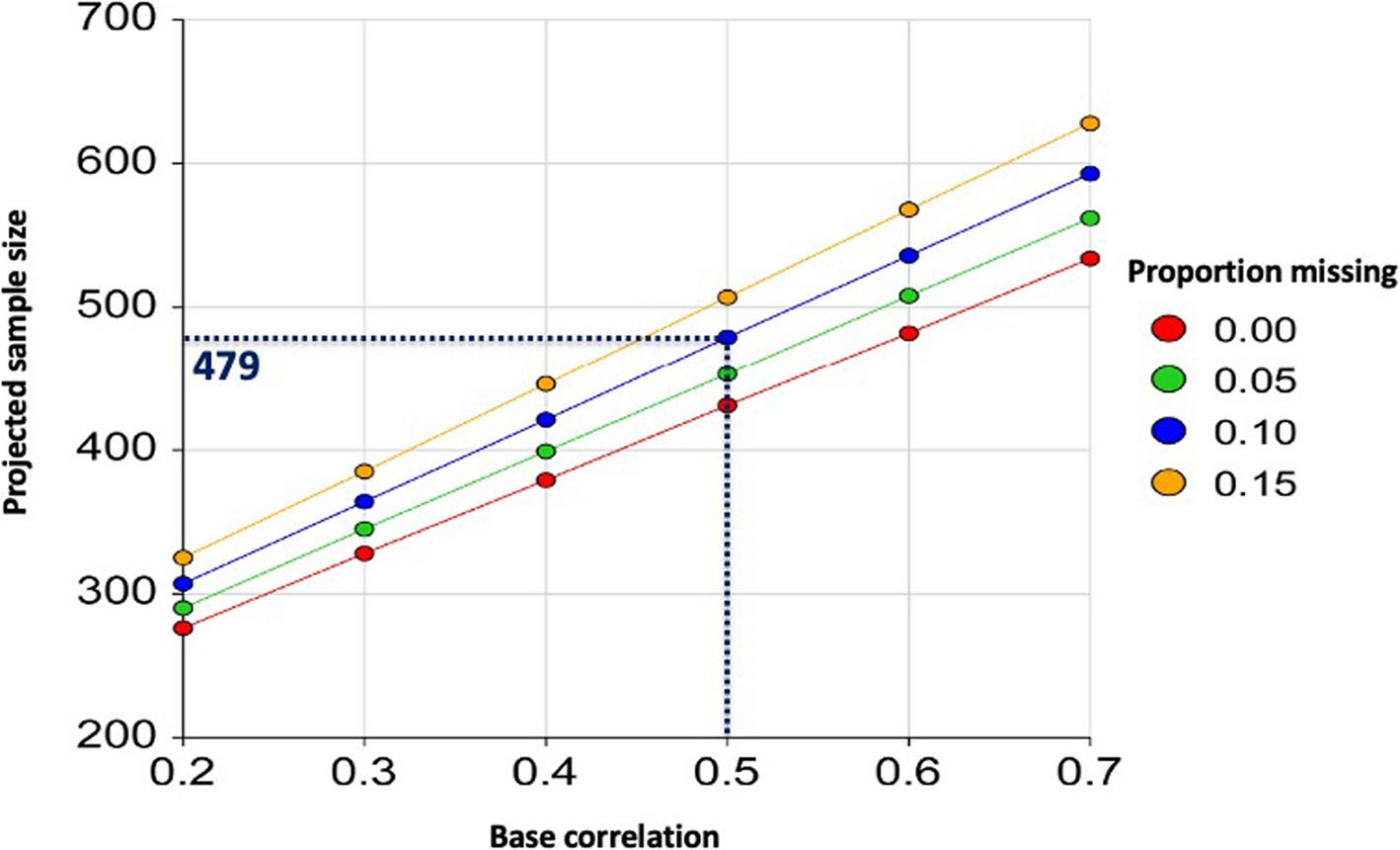
Projected sample size calculation using a generalized estimating equation approach with at least four repeated measures of the primary outcome, according to varying base correlation of 0.2 to 0.7 (between repeated measures) and degree of missingness of 0.0 to 0.15. A sample size of 479 donor/recipient pairs (1:1 allocation to maternal and paternal door kidneys, represented by discontinuous blue line) will be required to achieve a power of 80% with a two-sided significance of 5% for detecting a difference of 0.10 between marginal proportions after applying continuity correction (and correlations score of 0.5 of at least 4 observations [for the composite outcome measures], and accounting for potential missingness of data of up to 10%)

The sample size calculation is based on the primary composite outcome of de novo DSA, any episodes of acute rejection and allograft loss. In our ANZDATA registry study comparing the allograft outcomes of maternal and paternal donor kidney transplants, a greater proportion of kidney transplant recipients who had received maternal donor kidneys experienced any rejection episode compared with recipients of kidneys from paternal donors (37 and 27%, respectively; *p* < 0.001) [4]. Data relating to the development of de novo DSA are not available from ANZDATA registry. Assuming that up to 40% of recipients may develop de novo DSA, acute rejection or lose their allografts within 60 months post-transplant, a sample size of 479 donor/recipient pairs (1:1 allocation to maternal and paternal door kidneys) will be required to achieve a power of 80% with a two-sided significance of 5% for detecting a difference of 0.10 between marginal proportions after applying continuity correction (and correlations score of 0.5 of at least 4 observations [for the composite outcome measures], and accounting for potential missingness of data of up to 10%) (Fig. 2). The expected recruitment of 520 parental donor/recipient pairs in the retrospective study should have adequate sample size and sufficient power to address the study question. Sample size calculation was determined using the expected time-averaged difference (TAD) between two means from continuous, correlated data using the GEE method in PASS Sample Size Software.

Between 2015 and 2018, there were 182 pediatric and adolescent patients aged ≤18 years with kidney failure in Australia who received a kidney transplants, with parental donor kidney transplants contributing 40% of total transplants. This compared with 30 patients in New Zealand (67% parental donor kidney transplants) and 30 patients in the Netherlands (30-40% parental donor kidney transplants) in the same time-period. In the United Kingdom, there were 760 pediatric and adolescent patients who received kidney transplants between 2010 and 2015, with approximately 340 (45%) parental donor kidney transplants. These recent figures suggest that the target sample size (to recruit 520 donor/recipient pairs between 1990 and 2020) is achievable.

### Statistical analysis

Data will be presented as mean ± standard deviation (SD) or number (proportion) for continuous and categorical variables, respectively, with means and proportion compared using *t-test* and the chi-squared test where appropriate. We will develop models with different numbers of archetypes and choose which to use as the final model according to the residual sum of squares using the “elbow” method [47]. The archetypal models will assign scores based on a combination of pre-transplant immunological and clinical (donor, recipient and transplant) parameters to each recipient using the time from transplant to the composite primary outcomes of de novo DSA, any episodes of acute rejection and allograft loss; with the scores totalling 1. Each parameter will be assigned to a single archetype cluster on the basis of the highest archetype score (corresponding to “high immunogenic risk” profile, comprising of immunological risk factors). Additional models accounting for pre- and post-transplant factors (such as donor age, non-adherence) will also be constructed. A principal component analysis will be constructed to visualize the data matrix used as the input for the archetypal analysis. The principal component analyses will produce two main results: (i) a correlation circle, and (ii) a projection of the individuals. The correlation circle allows for a graphical examination of the relationships among the pre-transplant immunological and clinical parameters and the graphical parameter contribution of the axes (positive or negative contribution: vector direction; strength of the contribution: vector length when projected on the axis). We will identify distinct groups (i.e. archetypes), each comprising of a well-defined set of immunological and clinical parameters that may improve the risk stratification for “adverse immunological outcomes” for those accepting a parental donor kidney for transplantation, and separately for those who have received maternal or paternal donor kidneys. The Australian and New Zealand cohorts will be the derivation cohort for these archetypes, which will be validated in the cohorts from the United Kingdom and the Netherlands. We will next seek to build a predictive model (combined cohorts) to examine the associations between the archetype clusters and other pre-specified covariates and the primary outcome using univariate Cox proportional hazards regression models. A multivariable Cox model will be constructed by selecting covariates by Group Lasso and Doubly Robust Estimation (GLiDeR), a method of variable selection to identify confounders using an adaptive group lasso approach that simultaneously performs coefficient selection, regularisation, and estimation across the treatment and outcome models. Bootstrap resampling with replacement or subsampling without replacement will be used to investigate and quantify model stability. Separate models will be constructed for each of the secondary outcomes. The results of the model and covariates will be reviewed to ensure clinical relevance. Deviance and score residuals will be plotted to evaluate for poorly predicted individuals or individuals that may have had large influences on model parameters. The performance of the integrated prognostic model will be assessed by computing a non–time-specific area under the curve (AUC) using the measure of concordance from Somer’s Dxy statistic. For assessment of model calibration, patients will be risk-stratified on the basis of their predictive index, which is the linear combination of the product of the multivariate model β coefficients and their individual covariate values. Kaplan-Meier survival curves will then be plotted for patients who are stratified into quintiles of their predictive indices. The validation cohort’s AUC will be determined and predicted and observed mortalities will be compared. The association between archetypes and adverse outcomes will be examined in the prospective cohort, and to examine how the addition of other newly developed novel immunological assays will improve the test performance of the models.

## Discussion

The INCEPTION study including parental donor kidney transplant recipients across 4 countries reflects a study cohort of diverse ethnic distributions, which will allow for the generalizability of findings. The presence of local site study investigators and the implementation of site-specific study processes and procedures will ensure that we achieve the target recruitment. The investigators/authors (and delegates) of the protocol manuscript and appointed consumer and registry representative(s) of each country will form the steering and data monitoring committee, which will convene bi-annually and address issues relating to the conduct of the research and adherence to study protocol and processes. Any change in protocol or data capture will need to be approved by the steering and data monitoring committee prior to implementation.

### Storage of genetic materials and serum for related future research projects

With the current funding restrictions for this study, not all HLA and non-HLA genes or identification of anti-HLA and non-HLA antibodies will be possible. Genetic materials and sera obtained from patients (and corresponding donors) will be stored for a period of 15 years after completion of the initial study until future funding for additional projects become available. The steering and data monitoring committee of this study will provide oversight of potential research proposals regarding the use of these specimens and de-identified health information for future related projects.

### Confidentiality, data storage and record retention

All data will be managed in a confidential manner. Data will be stored on a secure server and only authorized investigators in the study team or their delegates will have access to the data. Health-related data will be retained for at least 15 years after completion of the project and relevant publications. The additional DNA and sera that are extracted/isolated and stored will be kept in a local or central facility, which will be pre-identified prior to commencement of study. If participants withdraw consent during the research project, the study doctor and relevant study staff will not collect additional information, although personal and other relevant information already collected will be retained to ensure that the results of the research project can be measured properly and to comply with law. Genetic materials and sera obtained for the purpose of this study will be kept locally or centrally for 15 years following completion of the initial study, after which the specimens will be destroyed and discarded appropriately.

### Dissemination of outcomes of project

It is anticipated that the results of this research project will be published and/or presented in a variety of forums, including national and international medical conferences. In any publications and/or presentations, only aggregate information will be provided in such a way that no participants can be identified. The results of these findings will also be disseminated through a newsletter summarising the salient findings to all study investigators, site investigators and other relevant people directly involved in the care of kidney transplant recipients. A summary of the study findings in lay person’s language will be disseminated to the participants.

The INCEPTION study findings will provide evidence to support or refute the apparent contradictory paradigm of differential outcomes associated with maternal versus paternal transplants, which can inform healthcare providers, clinicians, patients and their families of potential risks and expected long-term allograft outcome after accepting maternal compared to paternal donor kidneys. As children and adolescents have a higher need for maximizing transplant survival, the INCEPTION study is critical to supporting the evidence base to improve survival particularly for children and adolescents with kidney failure. In addition, kidney transplantation is associated with improved cognitive functioning [48, 49], health related quality of life [50], educational attainment and life participation [51]. Hence maximizing first allograft survival has ensuing benefits through increased social integration and labour productivity at the societal level. The INCEPTION study is important to improve the understanding of the immunological differences between accepting a kidney from the mother or father, which has not been possible until the recent development and availability of cutting-edge technology. This proposed program of work will enable an improved selection of the best available parental donor kidneys for children and adolescents with kidney failure, with subsequent improvements in the health outcomes for this at-risk population. There is considerable uncertainty regarding the utility, clinical application and significance of some of these methods and this study will systematically evaluate and validate the predictive power of combining the existing and novel pre-transplant B- and T cell molecular mismatch approaches to establish the influence of genetic compatibility in determining adverse allograft outcomes.

Additionally, this resource will further stimulate research interest in this and related areas leading to further improvements in kidney transplant and patient outcomes for this and other populations. Specifically, these include:

1. The predictive value of novel assessment of donor/recipient gene compatibility for adverse allograft and patient outcomes such as acute rejection, allograft loss, recurrence of primary kidney disease and other complications occurring after kidney transplantation.
2. The potential for individualizing immunological risk assessment to reduce adverse allograft outcomes in kidney transplant recipients.
3. Establishment of an important resource that will comprise the largest cohort of pediatric and adolescent kidney transplant recipients worldwide, with the availability of high-resolution HLA typing (using the most advanced typing technique in NGS sequencing) and complete allograft and patient data. The improvement of outcome in this cohort of patients is critical because of their projected lifespan, burden of chronic disease and the likelihood that these recipients will require repeat transplantation and continuing long-term utilization of healthcare resources in the treatment of their disease burden.

## Data Availability

Data sharing not applicable to protocol paper. Future study data will be presented within the manuscript and additional supporting files (where appropriate), but the availability of these data (in public repositories) will be according to each country-specific governance processes.

## Abbreviations

AMR: Antibody mediated rejection
AUC: Area under the cruve
ANZDATA: Australia and New Zealand Dialysis and Transplant
ANZCTR: Australian New Zealand Clinical Trials Registry
CADI: Chronic Allograft Damage Index
CKD-EPI: Chronic Kidney Disease-Epidemiology Collaboration
DSA: De novo donor-specific anti-HLA antibody
DNA: Deoxyribonucleic acid
EMS-3D: Electrostatic mismatch score
eGFR: Estimated glomerular filtration rate
GLiDeR: Group Lasso and Doubly Robust Estimation
HLA: Human leukocyte antigen
Ig: Immunoglobulin
MFI: Mean fluorescence intensity
NHMRC: National Health and Medical Research Council
NGS: Next generation sequencing
NIMA: Non-inherited maternal antigen
NIPA: Non-inherited paternal antigen
PRA: Panel reactive antibody
PAS: Periodic acid–Schiff
PIRCHE: Predicted indirectly recognizable HLA epitopes
SES: Socioeconomic status
STROBE: Strengthening the Reporting of Observational studies in Epidemiology
TG: Transplant glomerulopathy
UK: United Kingdom
